# Supplementary material for: Metabolic Fingerprints of Effective Fluoxetine Treatment in the Prefrontal Cortex of Chronically Socially Isolated Rats: Marker Candidates and Predictive Metabolites
Source: Int J Mol Sci. 2023 Jun 30;24(13):10957. doi: 10.3390/ijms241310957 (PMC10341512; doi:10.3390/ijms241310957)
Supplement: Supplementary file 1 [file ijms-24-10957-s001.zip › Supplementary Material S1.pdf]

Supplementary material for the part 4.7. SVM-LK-based binary classification

In the outer loop of cross-validation (CV2) the data were separated in 3 partitions named folds. 2 out of 3 folds were inserted in the inner loop of cross-validation (CV1) as training set. In the inner loop of cross-validation (CV1), the training set was separated again in 3-folds, 2 of them were used as input to the training process. The parameters of the model and the selected features were selected on the training set of CV1 and validated on the fold that was excluded. The selection of the parameters was based on the accuracy provided using linear binary SVM classifier (LIBSVM). After the parameter selection, the model was ready for testing on the fold that was left out of training on CV2. The average decision value in each of the binary CV1 ensembles was calculated in order to determine the group membership (average decision value > 0 or < 0) of the respective CV2 test subjects. The average accuracy, after 10 permutations in each loop, of all 900 models was extracted.

In detail, a two-step pre-processing procedure was applied:

a) prune non-informative columns of the matrix: if the values of a feature do not change between participants, the feature is excluded from the analysis.

b) scale the data from 0 to 1: transform each feature by linearly mapping its values into the range from 0 to 1.

In the feature selection process, greedy forward feature selection to select feature combinations that maximize the predictive accuracy of a model in the CV1 test data was applied, stopping at 50% of features and evaluating each feature. We classified a) CSIS without fluoxetine treatment vs. no CSIS and b) CSIS with vs. without fluoxetine treatment. SVMs are searching for the best boundary to separate the data into different hyperplanes, by maximizing the margin between the two groups and specifying the optimal hyperplane by the points lying on the margin boundaries called support vectors (Statnikov A, 2019).

After the pre-processing and the nested cross validation pipeline, the main step is the cross - validated regularization of the SVM-LK. L1 regularization sets the weights of non-informative data features to zero, allowing only essential and valuable data feature effects to be included into the machine learning model. We used L1 regularization, and cross-validation was used to select the C parameter that reflects the degree of penalty on a misclassified case. In order to avoid information leakage and create a generalized model for different samples, optimal values of C parameter are selected within the range of 2.^[-8:2:8] for each fold by validating 9 different models in total on the CV1 test set. The balanced accuracy of each model for every CV1 test set is calculated and the model with the best-balanced accuracy is finally selected and applied to the CV2 test set.

Statnikov, A.; Aliferis, C.F.; Hardin, D.P.; Guyon, I. A Gentle Introduction to Support Vector Machines in Biomedicine: Volume 2: Case Studies and Benchmarks; World Scientific Publishing Co., 2013; ISBN 9789814335157.
