# Supplementary figures and images for: Metabolic Fingerprints of Effective Fluoxetine Treatment in the Prefrontal Cortex of Chronically Socially Isolated Rats: Marker Candidates and Predictive Metabolites
Source: Int J Mol Sci. 2023 Jun 30;24(13):10957. doi: 10.3390/ijms241310957 (PMC10341512; doi:10.3390/ijms241310957)

A

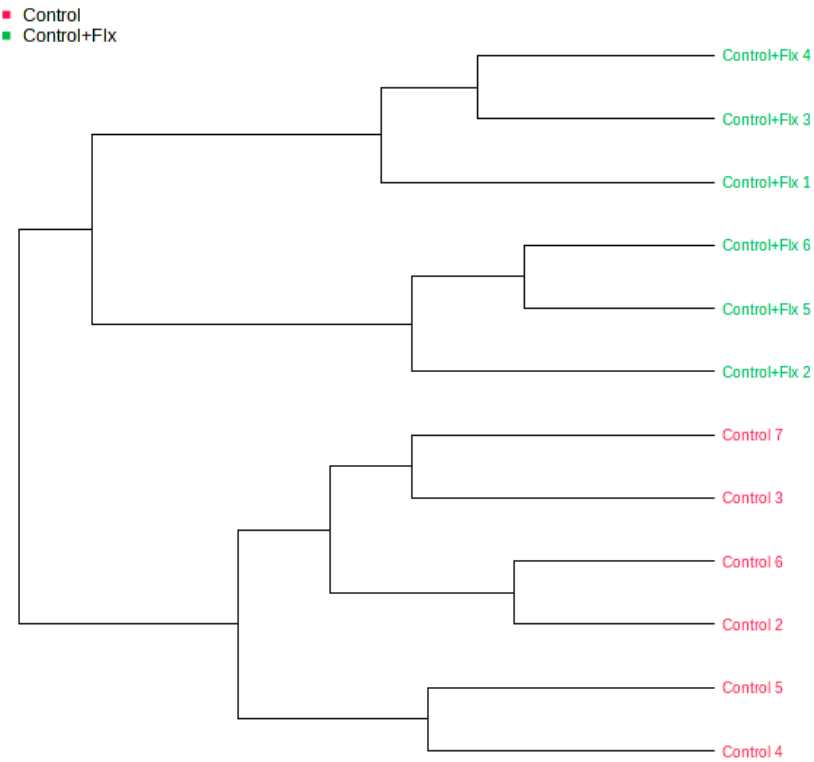

B

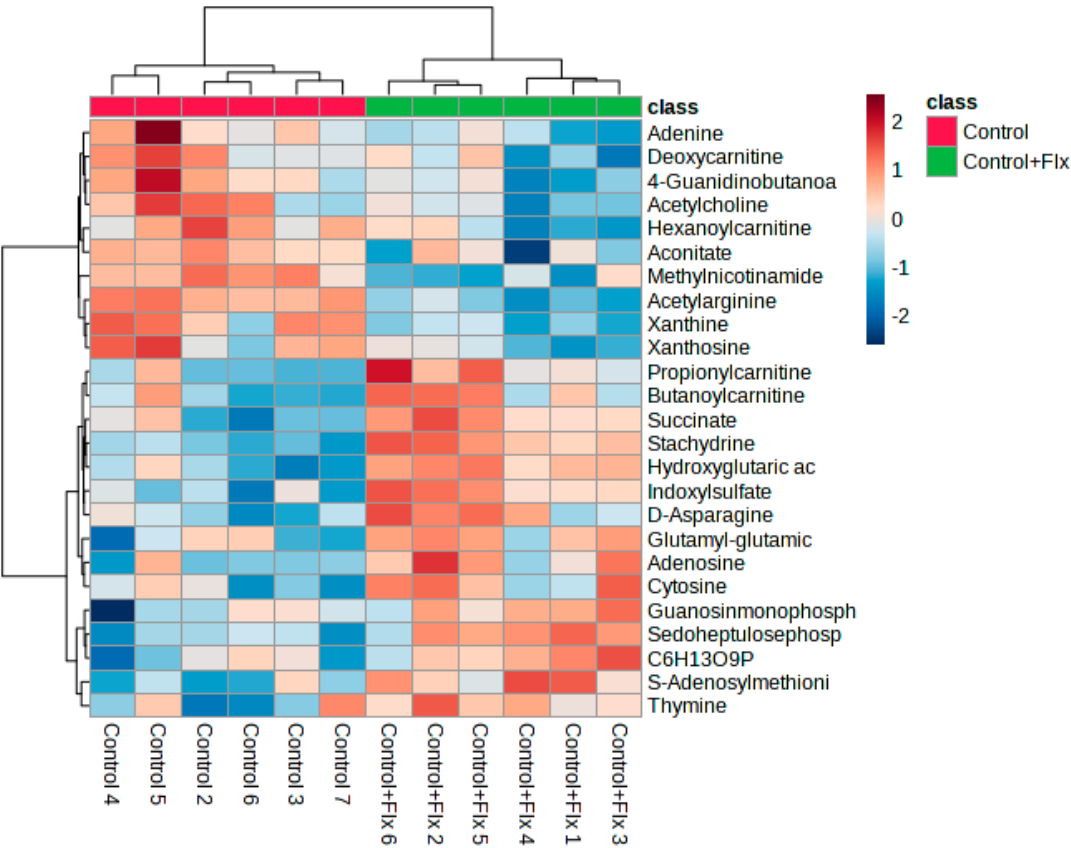

Supplement: Supplementary file 1 [file ijms-24-10957-s001.zip › Supplementary Figure S1.pdf]

A

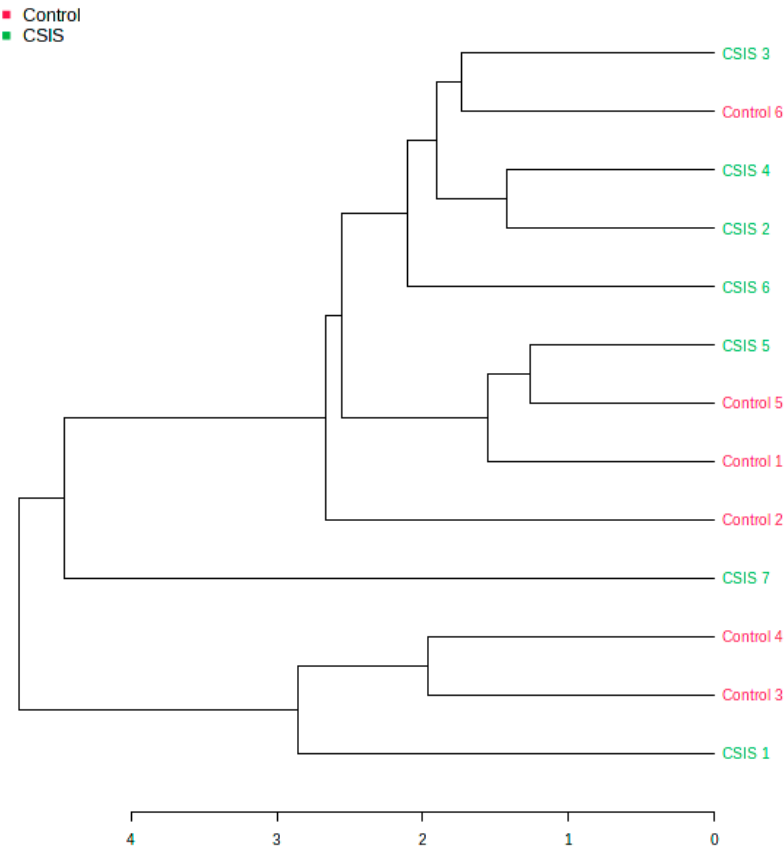

B

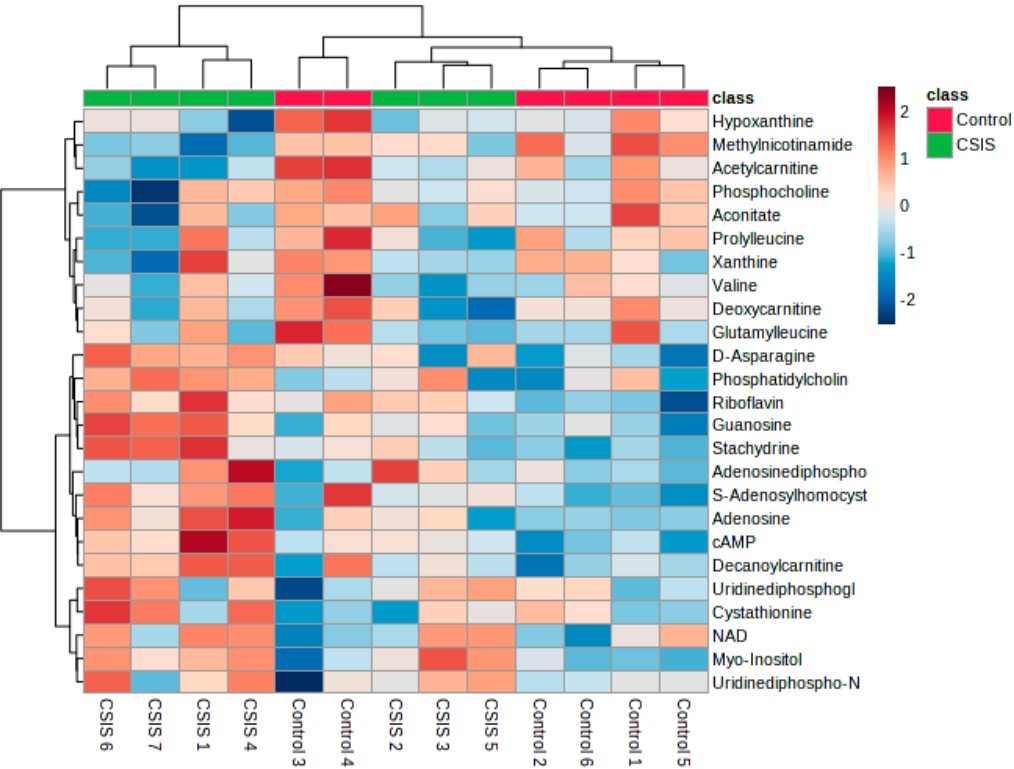

Supplement: Supplementary file 1 [file ijms-24-10957-s001.zip › Supplementary Figure S2.pdf]

A

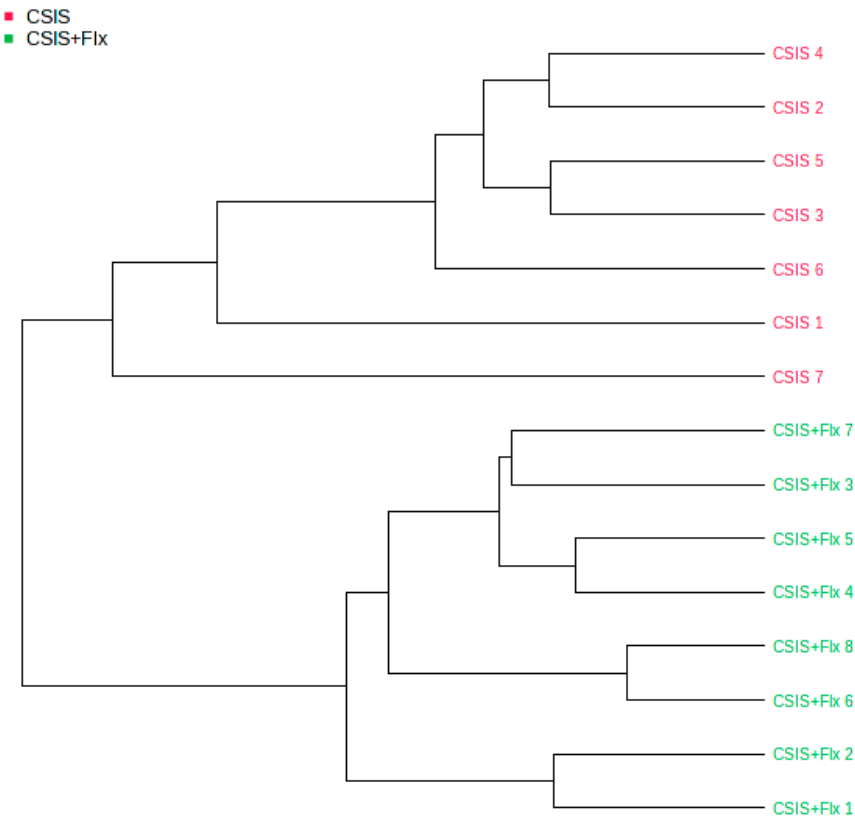

B

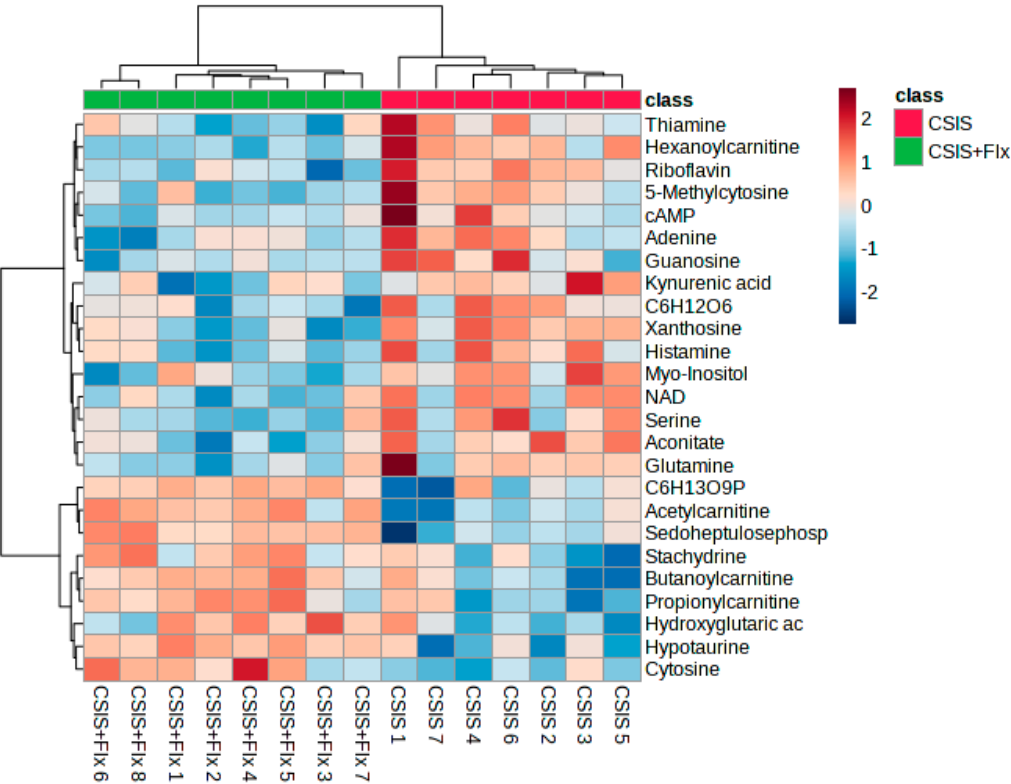

Supplement: Supplementary file 1 [file ijms-24-10957-s001.zip › Supplementary Figure S3.pdf]
